# Clinical implications of teleradiology in rheumatic and musculoskeletal diseases: improving rheumatic care

**DOI:** 10.1007/s00296-025-05810-w

**Published:** 2025-02-13

**Authors:** Yerlan Yemeshev, Bekaidar Nurmashev, Olena Zimba, Burhan Fatih Kocyigit

**Affiliations:** 1 Radiology Department, South Kazakhstan Medical Academy, Shymkent, Kazakhstan; 2 Department of Biology and Biochemistry, South Kazakhstan Medical Academy, Shymkent, Kazakhstan; 3 Department of Rheumatology, Immunology and Internal Medicine, University Hospital in Krakow, Krakow, Poland; 4 National Institute of Geriatrics, Rheumatology and Rehabilitation, Warsaw, Poland; 5 Department of Internal Medicine N2, Danylo Halytsky Lviv National Medical University, Lviv, Ukraine; 6 Department of Physical Medicine and Rehabilitation, University of Health Sciences, Adana City Research and Training Hospital, Adana, Türkiye

**Keywords:** Telemedicine, Teleradiology, Osteoarthritis, Osteoporosis, Rheumatoid arthritis, Spondyloarthritis, Rheumatic diseases, Surveys and questionnaires

## Abstract

Teleradiology, the transmission of radiologic images for remote assessment and consultation, has transformed modern medical care by mitigating geographical inequities and improving diagnostic accuracy. This technology employs telecommunications, digital imaging, and data-sharing systems developments to deliver swift and precise image analysis across various healthcare environments. Teleradiology has been essential in identifying and controlling diseases, including osteoarthritis, osteoporosis, rheumatoid arthritis, and spondyloarthritis, especially in musculoskeletal radiology and rheumatology. The combination of teleradiology and telemedicine has transformed multidisciplinary cooperation, enhancing communication among radiologists, rheumatologists, and other healthcare practitioners to provide patient-centered treatment. It has markedly enhanced access to highly specialized knowledge, especially in rural and disadvantaged areas, facilitating prompt consultations and alleviating patient travel constraints. However, despite its benefits, teleradiology encounters several challenges, including standardization issues, ethical dilemmas, and infrastructure constraints. The absence of uniform standards and inequalities in access to high-speed Internet and digital health records impede extensive implementation. Addressing these constraints is crucial to fully utilizing teleradiology’s potential in musculoskeletal and rheumatic care. This article highlights the transformational potential of teleradiology and its incorporation into telemedicine for musculoskeletal and rheumatological treatment. Teleradiology is set to enhance global healthcare delivery by addressing disparities in healthcare access, fostering multidisciplinary cooperation, and utilizing advanced technologies. It underscores the necessity for ongoing innovation and investment in infrastructure, education, and standards to optimize the advantages of this crucial technology and guarantee equitable, efficient, and high-quality care for all patients.

**Supplementary Information:**

The online version contains supplementary material available at 10.1007/s00296-025-05810-w.

## Introduction

**Fig. 1:**
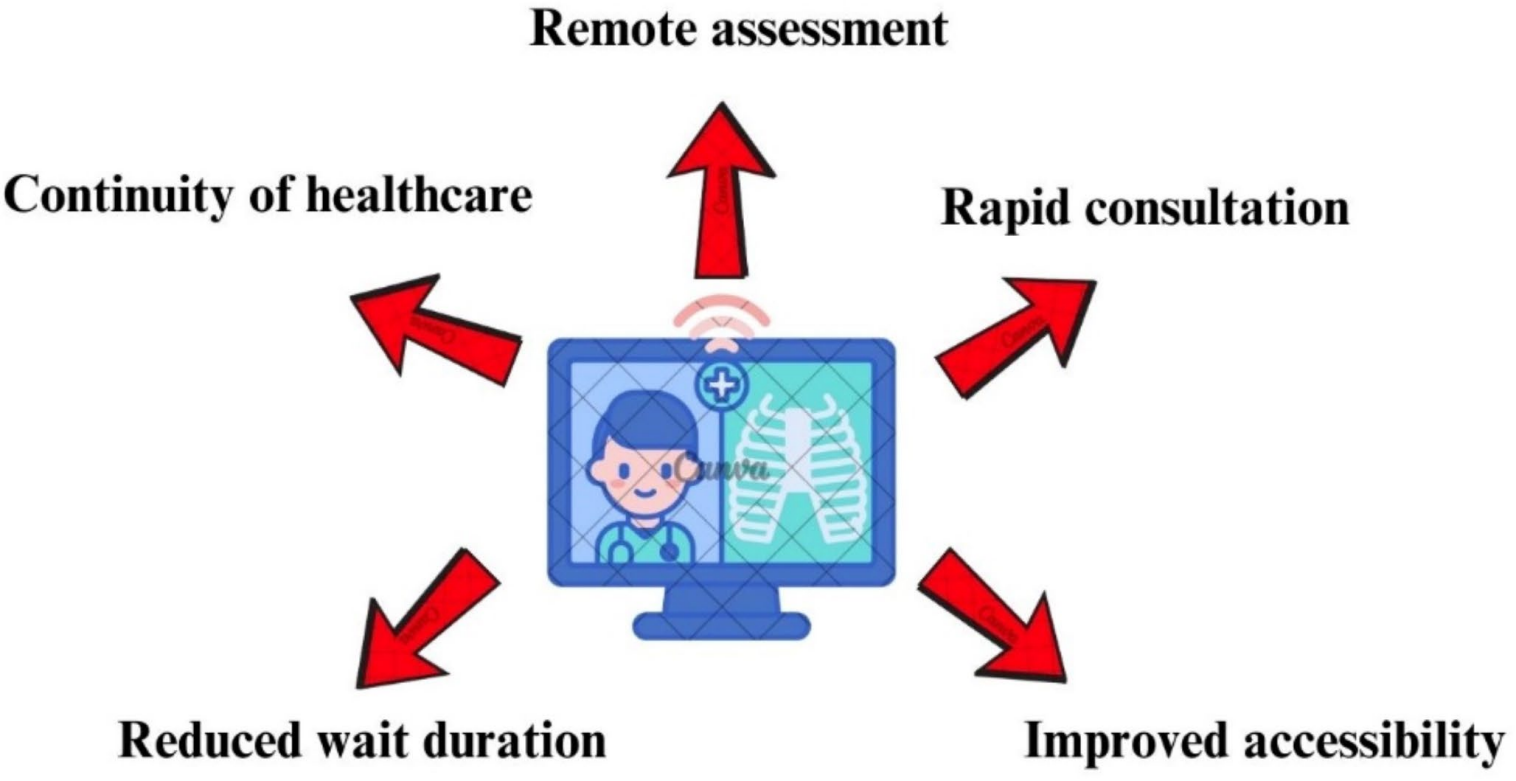
Main benefits of teleradiology

Teleradiology, the transfer of radiographic images and related healthcare information for remote assessment and consultation, has transformed healthcare imaging services globally. Teleradiology utilizes developments in digital image processing, telecommunications, and safe data-sharing systems to facilitate prompt diagnosis and medical care, particularly in areas with restricted access to radiologists [1]. This technology supports easy integration of imaging facilities across several sites, allowing healthcare organizations to operate more effectively. It serves a vital function in overcoming geographical obstacles and assisting healthcare professionals in emergencies, catastrophe contexts, and standard clinical procedures [2, 3] (Fig. 1).

**Fig. 2:**
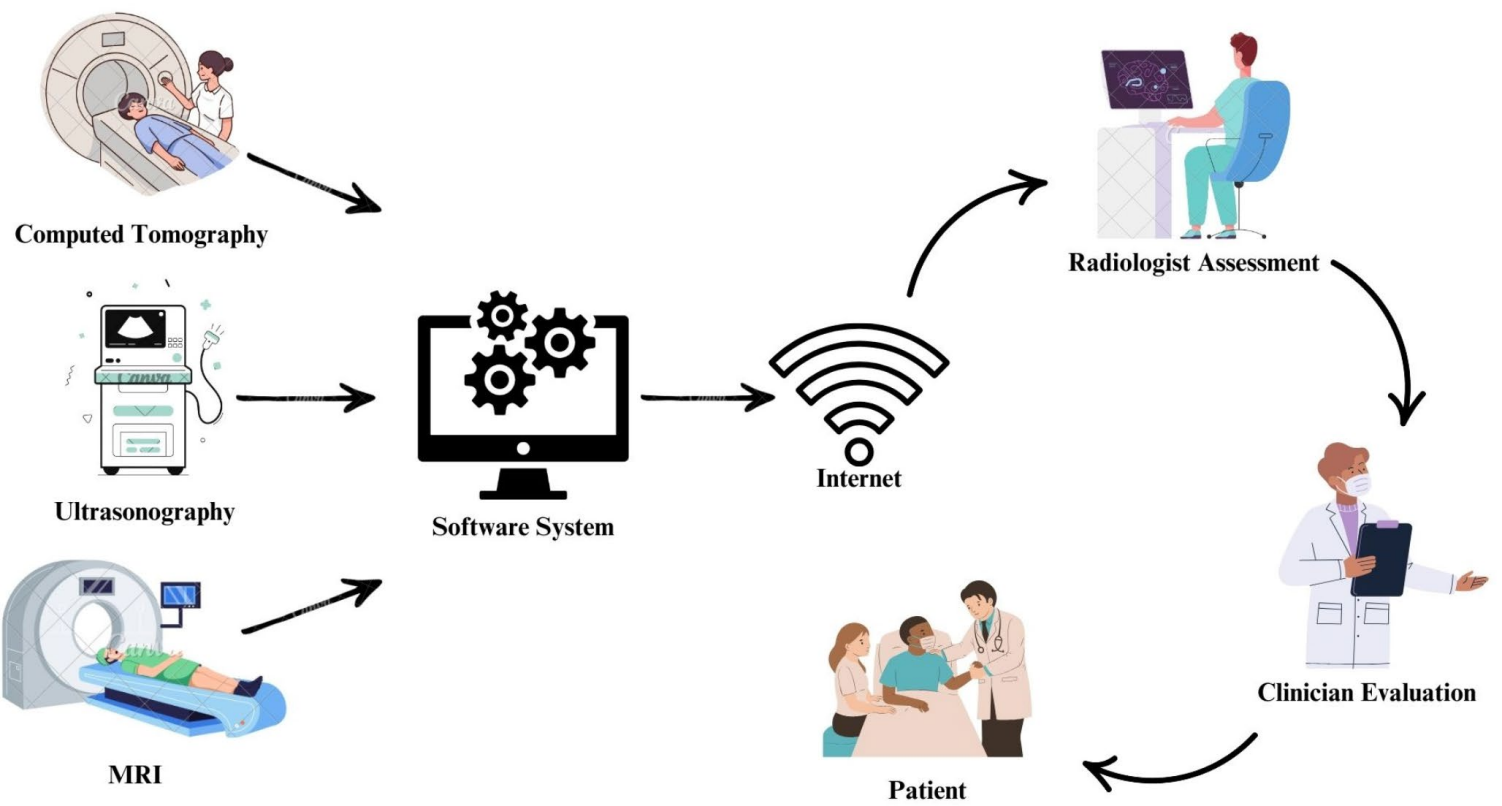
Teleradiology schema of operation

Teleradiology’s importance became particularly evident during the peripandemic phase, as it tackled distinct issues caused by the COVID-19 crisis. Amidst hospitals inundated with critically ill patients, teleradiology played a crucial role in ensuring the continued availability of medical imaging [4]. Radiologic instruments involving computed tomography (CT), magnetic resonance imaging (MRI), and ultrasound have been extensively employed to assess complications and other issues related to COVID-19 [5]. Teleradiology has facilitated radiologists’ remote image interpretation, ensuring continuous operations notwithstanding extensive quarantines and personnel deficits (Fig. 2).

The pandemic highlighted the value of adaptation and innovation in healthcare provision. Teleradiology emphasizes the necessity for resilient information technology structures, efficient communication systems, and standardized procedures to manage increases in imaging traffic [6]. In the post-pandemic period, teleradiology is increasingly stressing the integration of artificial intelligence, expanding interoperability, and providing access to professional imaging knowledge in underserved regions [7, 8]. These innovations are poised to transform the future of radiology, highlighting adaptability, effectiveness, and equity in medical provision.

Teleradiology substantially affects rheumatology, a discipline that frequently uses imaging modalities, including X-rays, ultrasound, and MRI, to diagnose and manage musculoskeletal issues. Teleradiology provides remote contact with radiology professionals, facilitating prompt evaluations of rheumatic disorders [9]. Incorporating teleradiology in rheumatology highlights its capacity to elevate patient care by facilitating enhanced access and collaboration between medical professionals [10].

## Aim

This article examines teleradiology’s transforming role in modern healthcare, focusing on musculoskeletal radiology and rheumatology. It aims to assess how teleradiology has enhanced diagnosis quality, facilitated timely consultations, and increased access to specialized imaging knowledge, particularly in underserved regions. By analyzing these achievements, the paper highlights the potential of teleradiology for transforming patient care in musculoskeletal and rheumatologic healthcare settings.

## Search strategy

A thorough search plan was set up before the literature review, and it was developed in accordance with the investigation methods provided by Gasparyan et al. [11]. During the preliminary phase of the investigation, the search phrase pairs were determined. The specified search terms were the following: ‘Telemedicine and Rheumatic Diseases’ or ‘Telemedicine and Musculoskeletal Diseases’ or ‘Telemedicine and Osteoarthritis’ or ‘Telemedicine and Osteoporosis’ or ‘Telemedicine and Rheumatoid Arthritis’ or ‘Telemedicine and Spondylarthritis’ or ‘Telemedicine and Surveys and Questionnaires’ or Teleradiology and Rheumatic Diseases’ or ‘Teleradiology and Musculoskeletal Diseases’ or ‘Teleradiology and Osteoarthritis’ or ‘Teleradiology and Osteoporosis’ or ‘Teleradiology and Rheumatoid Arthritis’ or ‘Teleradiology and Spondylarthritis’ or ‘Teleradiology and Surveys and Questionnaires’. MeSH terms were given precedence in the selection of combinations. The study incorporated publications from the Web of Science, Scopus, PubMed/MEDLINE, and the Directory of Open Access Journals (DOAJ). No inclusive timeline was determined. Additionally, researchers extensively reviewed the references in the publications and selected those deemed pertinent. Efforts were undertaken to prioritize the inclusion of more up-to-date content. Focused only on English articles.

## Teleradiology in osteoarthritis and osteoporosis

Osteoarthritis is a prevalent degenerative joint disease that dramatically affects quality of life. Telemedicine and teleradiology can be beneficial in diagnosing, monitoring, and managing this condition [12, 13]. Imaging techniques, including X-rays, MRI, and ultrasound, are essential for evaluating joint space narrowing, cartilage degeneration, and other structural alterations—critical evidence of disease progression. Teleradiology allows the transmission of imaging tests to distant specialists for prompt and precise interpretation, hence aiding in early diagnosis and tailored management strategies [14]. Tele-ultrasound has been utilized for remote educational and training purposes. A beginner ultrasound operator can be proficiently directed by a remote competent radiologist via particular ultrasound standards, letting the radiologist assess the diagnostic value of the obtained information remotely [15]. Teleradiology facilitates the transmission of medical images to distant specialists for prompt and precise interpretation, hence promoting early detection and tailored treatment strategies. This mainly benefits patients in rural or underdeveloped areas, where access to musculoskeletal imaging expertise may be restricted [16]. Teleradiology enhances multidisciplinary treatment by encouraging effective communication among radiologists, rheumatologists, physical medicine and rehabilitation specialists, and orthopedic surgeons [17].

Telemedicine and teleradiology have emerged as a critical instrument in assessing and treating osteoporosis, a disorder defined by diminished bone density and heightened fracture susceptibility [18]. Imaging modalities, including dual-energy X-ray absorptiometry (DEXA) and vertebral fracture assessments (VFA), are essential for diagnosing osteoporosis and evaluating treatment effectiveness [19]. Teleradiology provides online interpretation of imaging results, ensuring prompt identification of at-risk patients and encouraging early action. This is especially advantageous in areas where access to specialized bone health services is restricted. Teleradiology facilitates fracture risk evaluation by combining imaging data with clinical instruments like the FRAX outcome, thoroughly examining patient risk characteristics. With the growing integration of teleradiology in healthcare systems, using innovative image analytics and artificial intelligence will augment the identification and tracking of osteoporosis, thereby enhancing patient results and alleviating the burden of fragility fractures [20–22].

## Teleradiology in rheumatoid arthritis

Imaging modalities are crucial for identifying early indicators of joint degeneration, synovitis, and bone damage, which are vital for commencing prompt treatment and reducing disease progression. Moreover, these methodologies are important in illustrating the advancement of structural damage [23, 24]. Teleradiology permits remote access to professional radiographic interpretations, facilitating accurate evaluations and aiding in the formulation of tailored treatment strategies. Salaffi et al. [25] examined the efficacy of an intensive management approach utilizing a telemonitoring procedure compared to a standard care strategy in attaining remission and comprehensive control of the condition following one year. Hand and foot radiographs were assessed using a teleradiology approach and scored for radiological progression as part of the telemonitoring strategy. The findings underscored the benefits of the intensive telemonitoring approach compared to routine care for radiological progression. This research is relevant as it illustrates the efficacy of telemonitoring applications and confirms that radiologic progression can be evaluated and quantified remotely. Okino et al. [26] created in-house software featuring partial image phase-only correlation that can autonomously estimate the progress of radiologic joint space changes. The software’s efficacy on radiographs was juxtaposed with the joint space width variation, utilizing a micrometer as the benchmark for accuracy. The software assessed the radiographic progress of the finger joints from basal to the 52nd week. The cases were assessed using a standard visual scoring technique. The findings indicated that software can autonomously and accurately identify minor radiographic alterations in joint space width among individuals with rheumatoid arthritis. Incorporating comprehensive imaging algorithms and artificial intelligence in teleradiology can enhance the sensitivity and specificity of rheumatoid arthritis diagnosis, facilitating more accurate and effective treatment of the disease [27].

## Teleradiology in spondyloarthritis

Spondyloarthritis is a prevalent inflammatory rheumatic disorder. The delay in diagnosis remains a significant difficulty, persisting for an unpleasant duration of around 7 years. Neglected illness deteriorates prognosis, diminishes quality of life, and results in functional impairment and economic damage. The growing lack of rheumatologists and rising demand are expected to exacerbate diagnostic difficulties [28, 29]. Hannah et al. [30] performed a telemedicine investigation in patients with spondyloarthritis, revealing a notable enhancement in diagnostic sensitivity with the availability of imaging information. Relying solely on personal health-related history data, including symptom checklists, demonstrated that expert rheumatologists attained a diagnosis certainty of merely 27% [31]. In a comprehensive video consultation diagnosis investigation, the precision was significantly superior in an approach predominantly based on radiology and laboratory information, in contrast to methods that extensively depended on physical examination [32]. The constraints of physical examination during video consultations hinder the correctness of remote diagnosis. The growing accessibility of teleradiology methods and novel smartphone-based technologies progressively alleviate these constraints [30]. Teleradiology allows remote specialists to interpret imaging findings, facilitating prompt diagnosis and treatment, especially in underprivileged regions with restricted access to rheumatology expertise. This method not only assists in identifying early indicators of disease but also provides continuous observation of disease progress. Developments in artificial intelligence and computerized image processing enhance teleradiology, facilitating the discovery of subtle imaging results and promoting more effective and tailored medical care for patients [33].

## Overview of surveys on teleradiology

Survey-based research on teleradiology offers essential information regarding its acceptance, efficacy, and influence on patient outcomes. These studies frequently concentrate on factors such as user satisfaction, diagnostic precision, and obstacles to adoption [34]. Patient-centered surveys can yield valuable data regarding their knowledge, attitudes, and behaviors.

A survey of radiologists in the United States revealed that 77.7% are currently engaged in teleradiology within their practice. Furthermore, an extra 9.4% indicated that they had engaged in such activities within the last decade. An overall percentage of 76.9–86.2% of respondents recognized the value of teleradiology in terms of geographical, after-hours, and multispecialty coverage, along with decreased interpretation turnaround times. The main obstacles faced in teleradiology include the lack of reaching digital health records (62.8%), ensuring quality (53.8%), and the proximity of technological experts (48.4%) [35].

Alruwaili et al. [36] conducted a nationwide survey-based investigation regarding teleradiology in Saudi Arabia. More than 95% of the participants concurred that teleradiology programs proficiently resolve medical inquiries, demonstrating a substantial agreement on the clinical efficacy of this method. 90% of the respondents expressed satisfaction with teleradiology assistance, reflecting a favorable perception of the quality and effectiveness of the services. A considerable number of respondents (%93) acknowledged the necessity of standardizing MRI techniques among healthcare facilities, underscoring the shared acknowledgment of the advantages linked to protocol uniformity. The primary advantages of teleradiology were as follows: improvements in radiologist reporting facilities (73.2%), significant efficiency advancements in routine tasks (64.6%), availability of second views (64.6%), and reductions in reporting time (53.7%).

A survey-based investigation of telemedicine availability in Europe revealed that teleradiology was covered in 74% (28 out of 38) of the nations surveyed. Among the member states that responded to the inquiry regarding teleradiology’s maturity level, 53% reported establishing teleradiology operations. Additionally, 31% were conducting running experiments or providing teleradiology for unofficial operations [37].

In the study examining telesonography, the rating scores indicated that all radiologists classified patient communication and imaging quality as “very good.” Three participants (60%) assessed ergonomics as “very good,” whilst two participants (40%) evaluated convenience as “good”. According to the radiologist’s blinded evaluation, the visual representation of structures in the body was comparable among robotic and conventional visuals [38].

A survey-based study examining patient preferences and feedback concerning teleradiology revealed that participants expressed high satisfaction with remote visual comprehension, the level of care, comprehension, and simplicity of use. The main obstacles cited by patients included technological challenges, feedback issues, concerns regarding privacy, and insufficient awareness [39].

## Perspectives on teleradiology

### Integration of artificial intelligence and novel technologies in teleradiology

Artificial intelligence and machine learning have contributed to substantial progress in teleradiology, especially in musculoskeletal radiology and rheumatology. Artificial intelligence-driven processes facilitate automated image analysis, decreasing interpretation duration, augmenting diagnostic precision, and optimizing workflow productivity [40]. These tools support radiologists in identifying early indicators of disorders such as osteoarthritis, osteoporosis, and inflammatory joint diseases, which may be subtle or neglected in manual evaluations.

Recent advancements in deep learning models have exhibited enhanced efficacy in detecting musculoskeletal disorders, particularly during the evaluation of X-rays, MRI, and ultrasound images. Artificial intelligence-driven systems can differentiate between inflammatory and degenerative joint diseases with considerable sensitivity and specificity, facilitating early intervention and tailored treatment strategies [41–43]. Besides diagnosis, artificial intelligence is important in optimizing radiology workflows by [44, 45]:

- *Automated Triage and Prioritising*: Artificial intelligence can identify urgent situations, guaranteeing that radiologists swiftly assess critical observations (e.g., fractures, inflammatory alterations, or malignancies).
- *Natural Language Processing*: Artificial intelligence-powered solutions help to generate organized radiology reports, improve uniformity, reduce reporting errors, and facilitate interactions between radiologists and referring physicians.
- *Quantitative Imaging Analysis*: Artificial intelligence facilitates accurate measures of bone density, joint space narrowing, and damage progress, allowing for more reliable evaluations in rheumatic disorders.

Expanding artificial intelligence integration in teleradiology has substantial potential to boost diagnostic accuracy, alleviate workload pressures, and improve patient outcomes, particularly in musculoskeletal and rheumatic care. Future research needs to concentrate on enhancing artificial intelligence algorithms to facilitate their smooth integration into practical therapeutic settings.

### Global accessibility of teleradiology

Teleradiology provides a revolutionary approach to address inequality in healthcare in poor regions where access to radiologists is frequently constrained. This approach facilitates remote interpretation of radiographic results, helping patients in rural and resource-limited regions to receive prompt and precise diagnoses without extensive travel [46]. Notwithstanding its potential, obstacles, including insufficient infrastructure, restricted internet connectivity, and elevated implementation costs, impede extensive adoption. Resolving these difficulties necessitates focused investments in telecommunication infrastructure, financial assistance for equipment, and creating scalable and cost-effective solutions [47]. The evolution of teleradiology highlights its capacity to democratize healthcare delivery, emphasizing its significance in fostering a more inclusive and efficient global healthcare system.

### Standardization and established protocols in teleradiology

Standardization guarantees that imaging examinations are conducted, processed, and sent consistently, regardless of equipment or location. This consistency is essential for preserving diagnostic accuracy and enabling radiologists to interpret images reliably, irrespective of their source. Established protocols enhance quality assurance, allowing healthcare providers to efficiently compare imaging data over time and between institutions [48]. Cooperative initiatives among medical professionals, technology developers, and regulatory authorities can facilitate the creation and execution of universal standards.

### Ethical and privacy concerns

Ethical privacy concerns are crucial in adopting and growing teleradiology, as the security of medical information is paramount. The Internet’s transmission of private imaging data presents potential breaches, unauthorized usage, and data exploitation issues. The increasing global flow of healthcare data needs more solid and effective legal frameworks to ensure equitable access and safeguard patient privacy. Countries exhibit diverse rules about data protection. Adherence to these frameworks is crucial for the ethical utilization of patient data. The absence of harmonization among foreign regulations presents issues for global teleradiology networks [49]. Moreover, worries around data ownership, patient consent, and third-party access compound the ethical complexities. Who possesses the imaging data after it is transmitted between healthcare systems? Are patients permitted to decline remote storage and analysis of their imaging records? These questions necessitate standardized legal frameworks and strong governance systems to uphold patient trust. Patients may be concerned about managing their personal health information, particularly when services traverse national borders with varying legislation. Progress in safe data-sharing techniques is mitigating these issues by applying powerful encryption techniques, blockchain solutions, and secure cloud infrastructures [50, 51].

### Practical implications and messages

The present article offers significant insights into the growing function of teleradiology, especially concerning musculoskeletal and rheumatic disorders. This article consolidates existing knowledge on teleradiology’s advantages, challenges, and prospects, serving as a valuable reference for healthcare providers, radiologists, and researchers interested in its integration into regular clinical practice.

- *Improved accessibility*: Teleradiology addresses the disparity in radiology services for underserved and remote areas, facilitating prompt diagnosis and treatment planning.
- *Effectiveness and workflow optimization*: Artificial intelligence-driven automation enhances reporting precision, diminishes interpretation duration, and prioritizes urgent instances for swift intervention.
- *Standardization and quality assurance*: Universal protocols improve diagnostic uniformity between institutions and healthcare environments.
- *Ethical and privacy aspects*: Robust data-sharing options are crucial for preserving patient confidentiality and adhering to legal regulations.
- *Future directions*: Further investigation is required to enhance artificial intelligence applications, resolve infrastructure constraints, and incorporate teleradiology effectively into musculoskeletal and rheumatic care.

This study provides a thorough and up-to-date review of teleradiology’s roles in musculoskeletal radiology and rheumatology, surpassing previous research in Rheumatol Int by incorporating recent developments in artificial intelligence, global accessibility issues, and standardization protocols. It underscores the transformative capacity of artificial intelligence-enhanced diagnostics and workflow efficiency, the urgent necessity for infrastructure enhancement in underprivileged areas, and the significance of universal standards for reliable diagnostic precision. Furthermore, it accentuates patient-centered outcomes and ethical implications, offering a comprehensive perspective on the evolving domain of teleradiology.

## Conclusion

Teleradiology has become an innovative feature in modern medical care, providing exceptional prospects to improve accessibility, increase diagnostic precision, and promote collaboration among medical specialties. Its enormous influence is especially apparent in musculoskeletal radiology and rheumatology, where disorders including osteoarthritis, osteoporosis, rheumatoid arthritis, and spondyloarthritis greatly benefit from its improvements. Teleradiology facilitates remote interpretation of imaging modalities such as X-rays, MRI, and ultrasound, ensuring prompt and accurate diagnosis, particularly in impoverished areas. Despite ongoing obstacles, including standardization, ethical issues, and infrastructural inequalities, progress in artificial intelligence, safe data-sharing technologies, and interoperability consistently improve its functionalities. By confronting these challenges and utilizing its capabilities, teleradiology is positioned to transform musculoskeletal and rheumatological care, guaranteeing equitable and efficient healthcare delivery globally.

## Electronic supplementary material

Below is the link to the electronic supplementary material.

Supplementary Material 1

Supplementary Material 2

Supplementary Material 3

Supplementary Material 4

Supplementary Material 5

Supplementary Material 6

Supplementary Material 7

Supplementary Material 8

## Data Availability

There is no stored data set associated with the article.

## Declarations

### Final approval

YY, BN, OZ, and BFK. All co-authors take full responsibility for the integrity and accuracy of all aspects of the work.

### Disclaimer

Authors state that no part of this manuscript, including the text and graphics, are copied or published elsewhere in whole or in part.
